# Clinical outcomes of using bilateral buccinator myomucosal flaps in cleft lip and palate patient with velopharyngeal insufficiency: case report

**DOI:** 10.1186/s40902-025-00464-x

**Published:** 2025-06-09

**Authors:** Tae Hyeong Park, Jin-A Baek, Seung-O Ko

**Affiliations:** Department of Oral and Maxillofacial Surgery, School of Dentistry, Jeonbuk National University Dental Hospital, Jeonju, Korea, Republic of

**Keywords:** Buccinator myomucosal flap, Velopharyngeal insufficiency, Speech aid, Cleft palate

## Abstract

**Background:**

Velopharyngeal insufficiency (VPI) occurs in 5–36% of patients after primary palatorrhaphy for cleft palate, causing hypernasality and nasal emissions due to inadequate velopharyngeal closure. Although various surgical treatments are available, they may present limitations and potential risks, including obstructive sleep apnea. The buccinator myomucosal flap, with a reliable blood supply, provides a versatile option for VPI correction. In addition, it is associated with a low risk of complications, further supporting its safety and applicability in clinical practice. We report successful palatal lengthening using a modified bilateral buccinator myomucosal flap with a buccal fat flap in a 14-year-old patient with persistent VPI despite prior interventions.

**Case presentation:**

A 14-year-old female with a bilateral complete cleft lip and palate underwent primary cheiloplasty at 3 months and palatorrhaphy at 9 months of age. Despite 10 years of speech therapy and 4 years of speech aid use, hypernasality persisted. To address this, palatal lengthening was performed using bilateral buccinator myomucosal flaps combined with buccal fat flaps. At 1 month postoperatively, partial necrosis of the buccal fat grafts was observed but healed without further complications. At 8 months postoperatively, soft palate elongation exceeding 1 cm was achieved, and nasometric assessments demonstrated nasality reductions of 25.5 percentage points for high vowels (/i/, /wi/) and 19.5 percentage points at the sentence level. In the consonant accuracy evaluation, the patient’s word-level accuracy increased from 72.09% preoperatively to 88.37% at 6 months postoperatively. These objective improvements correlated with subjective reports of improved speech and reduced vocal effort.

**Conclusion:**

As seen in this case, the combined use of buccinator myomucosal and buccal fat flaps can be a viable surgical option for addressing VPI through soft palate lengthening. This approach can lead to improvement in hypernasality with minimal complications, and its efficacy may be further supported by future long-term follow-up studies involving larger patient populations.

## Introduction

Cleft palate is one of the most prevalent congenital anomalies of the orofacial region globally [1]. Surgical correction of cleft palate helps prevent functional, social and psychological problems associated with deformity [2]. However, approximately 5–36% of patients undergoing primary palatorrhaphy experience velopharyngeal insufficiency (VPI) [3].

VPI occurs when the velopharyngeal valve fails to close properly during speech, breathing, and swallowing due to structural abnormalities in the soft palate and pharyngeal wall. The primary symptoms of VPI include hypernasality and nasal emission during speech, which result from insufficient closure between the nasal and oral cavities [4]. Consequently, VPI can impact not only speech intelligibility but also overall quality of life.

Treatment options for children with VPI include non-surgical methods and surgical interventions. Non-surgical methods include speech therapy combined with the use of speech prosthetics, such as palatopharyngeal obturators, palatal lifts, and pharyngeal bulbs. Surgical treatment is typically indicated for patients who are unable to use speech prosthetics or those who have not achieved sufficient improvement with non-surgical methods [5–7].

Surgical treatments for VPI include procedures such as V–Y pushback, pharyngeal flap, sphincter pharyngoplasty, and double reverse Z-plasty, among others. While satisfactory speech outcomes have been reported with these surgical interventions, their indications may be limited, and some are associated with complications such as obstructive sleep apnea, snoring, and mouth breathing [8–10].

The buccinator myomucosal flap is one of the surgical options employed to treat VPI by extending the palate. Initially described by Bozola et al. [11] in 1989, this flap is vascularized by the buccal artery and the posterior branches of the facial artery. Its reliable blood supply enhances its safety and makes it a versatile option for reconstructing various intraoral structures [12–14]. This approach has been reported to carry a lower risk of complications, such as snoring, obstructive sleep apnea, and mouth breathing, compared to palate-lengthening procedures such as pharyngeal flap and sphincter pharyngoplasty [10, 15]. In 2023, Kareem et al. introduced a modified bilateral buccinator myomucosal flap combined with a buccal fat flap technique. This approach involves making a full-thickness mucosal incision on the buccinator flap and covering the pedicle with a buccal fat flap. Additionally, buccal fat flaps can be extended to fill the intervening dead space between the nasal and oral myomucosal flaps [16].

This case report presents the successful application of modified bilateral buccinator myomucosal flaps combined with buccal fat flaps for palatal lengthening in a 14-year-old patient with persistent VPI, despite prior primary palatorrhaphy and the use of speech prosthetics. It aims to describe the surgical technique and assess outcomes, particularly regarding hypernasality improvement following the procedure.

## Case presentation

**Fig. 1:**
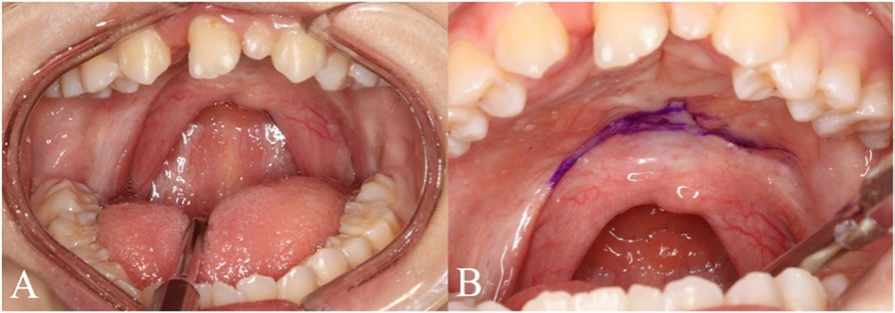
Pre-operation intraoral assessments. **A** Pre-operation intraoral examination presenting short soft palate.** B** Marking hard-soft palate junction

The patient is a 14-year old female with no significant medical history or syndromic features. She was diagnosed with a bilateral complete cleft lip and palate and was fitted with a Hotz plate at 2 weeks of age to assist with feeding. Primary cheiloplasty was performed under general anesthesia at 3 months of age, followed by palatorrhaphy using the V–Y pushback technique at 9 months. Despite undergoing speech therapy for 10 years, she continued to exhibit hypernasality, leading to inaccurate articulation. A speech aid had been worn for 4 years; however, hypernasality persisted. Speech evaluation revealed a consonant accuracy of 69.77% at both the word and sentence levels, with primary errors characterized by nasalized distortions of velar, alveolar, and palatal sounds. A short palate was observed on clinical examination. (Fig. 1) These findings suggested that the patient’s persistent hypernasality is primarily due to a short soft palate rather than inadequate muscle function. Based on this assessment, palatal lengthening with bilateral buccinator myomucosal flaps combined with buccal fat flaps was planned to address the hypernasality associated with VPI.

**Fig. 2:**
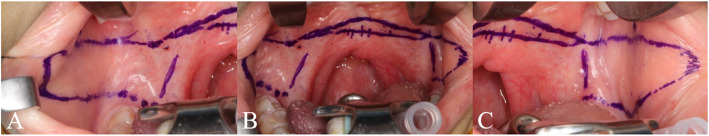
Flap design for bilateral buccinator myomucosal flap for palatal lengthening. **A**,** C** Bilateral buccinator myomucosal flap design. **B** Incision line at hard-soft palate junction

The surgery was performed under general anesthesia with orotracheal intubation, and the palatal area was exposed using a Dingman retractor. The hard-soft palate junction and bilateral buccinator myomucosal flaps were designed for palatal lengthening (Fig. 2).

The superior buccal incision was made just below the maxillary gingiva and extended in a straight line toward the lateral commissure, carefully avoiding Stensen’s duct. The inferior incision began at the retromolar trigone and extended in a curved fashion toward the lateral commissure. Both incisions were extended into the palatal defect to allow for sufficient mobilization and tension-free closure.

After flap elevation, the left buccinator myomucosal flap was folded into the defect, aligning the buccal mucosa with the nasal mucosa of the palate, and was sutured to ensure complete nasal mucosal closure. The flap was elevated by dissecting deep to the buccinator muscle while remaining superficial to the buccopharyngeal fascia, minimizing the risk of excessive buccal fat herniation.

**Fig. 3:**
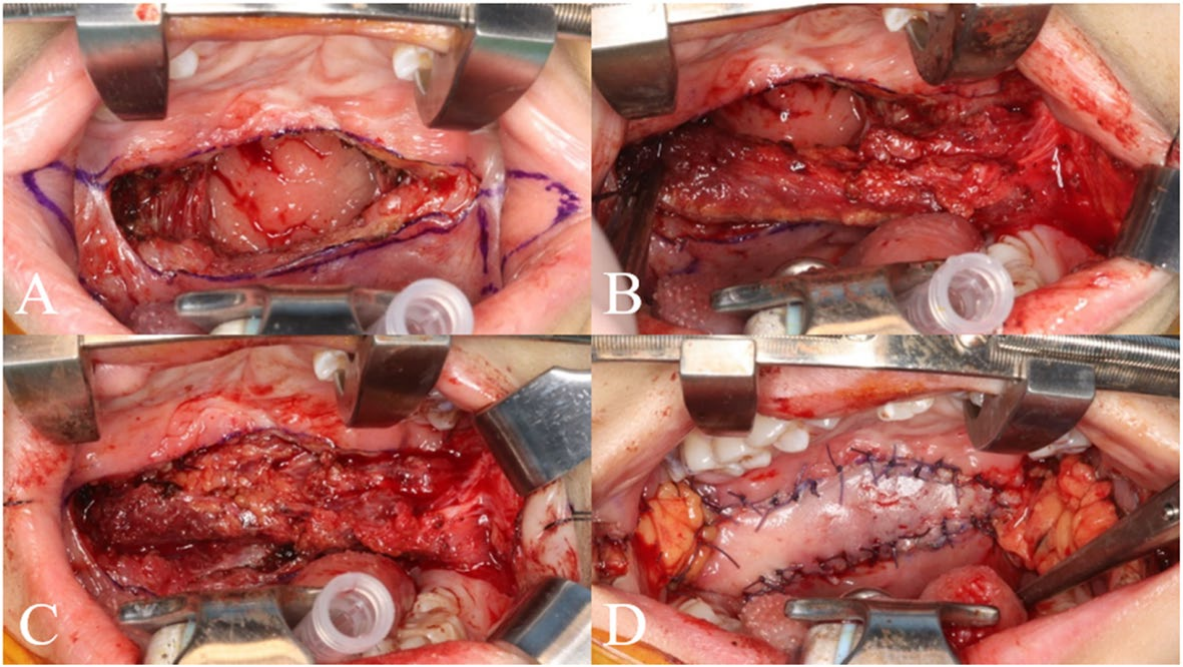
Surgical procedure of bilateral buccinator myomucosal flap with buccal fat flap for palatal lengthening. **A** Dissection of the soft palate exposing nasal mucosa. **B** Left buccinator myomucosal flap elevation. **C** Rotation and suture left bucccinator myomucosal flap and nasal mucosa. **D** Bilateral buccalf fat flap graft and suture right buccinator myomcosal flap at palatal lenthening area

Next, bilateral buccal fat flaps were elevated by opening the buccopharyngeal fascia anterior to the buccinator myomucosal flap pedicle on both sides, then advanced medially and sutured over the nasal myomucosal flaps. The contralateral buccinator myomucosal flap was rotated 180° clockwise and positioned over the buccal fat flaps to establish the oral mucosal layer. The donor site was sutured, leaving a small opening near the flap pedicle to relieve tension, while the base of the buccal fat flaps covered the pedicle of the buccinator myomucosal flaps, promoting mucosal ingrowth over the vascularized buccal fat. A spanning suture may be used to prevent buccal fat herniation (Fig. 3).

**Fig. 4:**
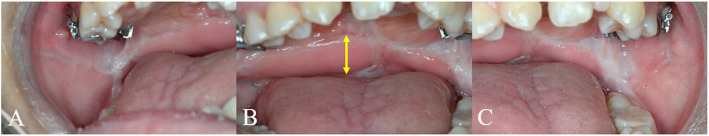
Post-operative 6 months follow-up. **A**,** C** Bilateral buccal fat flap sites. **B** Buccinator myomucosal flap graft site(yellow arrow: over 1 cm lengthening)

**Fig. 5:**
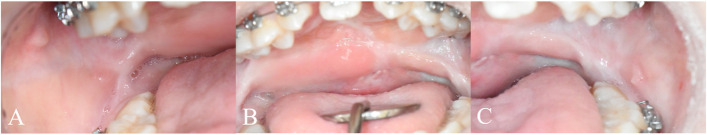
Post-operative 12 months follow-up. **A**,** C** Bilateral buccal fat flap sites. **B** Buccinator myomucosal flap graft site

A liquid diet was provided for 5 days postoperatively to prevent irritation at the surgical site, followed by the introduction of a soft diet on day 6. At the one-month postoperative follow-up, partial necrosis was observed at both buccal fat flap graft sites; however, the graft sites subsequently healed well without complications. The soft palate showed a lengthening of over 1 cm (Fig. 4). At the 12-month follow-up, both the donor and graft sites were well maintained without any surgical complications (Fig. 5). No postoperative complications such as snoring, mouth breathing, or obstructive sleep apnea were observed.

To evaluate improvements in speech outcomes, nasalance was assessed periodically using the Nasometer II™ (Model 6400, KAY Elemetrics Corp., Lincoln Park, NJ, USA), and articulation was evaluated by measuring consonant accuracy with the Urinal Test of Articulation and Phonology (U-TAP). Both assessments were conducted by a single speech therapist. At 8 months postoperatively, nasalance assessments showed an average improvement of 25.5 percentage points in nasality for high vowels (i, wi) and 19.5 percentage points for sentence-level nasality (Tables 1 and 2). In the consonant accuracy evaluation, the patient’s word-level accuracy increased from 72.09% preoperatively to 88.37% at 6 months postoperatively. Although a formalized self-evaluation scale was not used in this case, both the patient and her parents consistently reported improvement in hypernasality and reduced speech effort during follow-up interviews.

**Table 1:** Nasalance comparison of vowels

	Pre-speech aid	Post-speech aid	Pre-operation	PDM 1M	POD 3M	POD 8M
a	20	15	30	36	19	17
i	73	69	79	79	55	49
e	30	27	42	49	32	29
o	42	48	62	56	34	40
u	61	51	74	73	46	40
ja	25	24	32	34	22	20
je	38	34	42	53	32	25
wi	56	59	67	74	46	46

Units: %

**Table 2:** Nasalance comparison of sentences

	Pre- speech aid	Post speech aid	Pre-operation	POD 1 M	POD 3 M	POD 8 M
Sea passage	30	30	35	48	22	20
Rabbit passage	41	38	54	54	27	30

Units: % Sea passage: “I’ll go to the beach on Monday afternoon to catch clams and shrimps, and come back early in the morning on Tuesday.” Rabbit passage: “Let’s open the book together. It is the running story of a turtle and a rabbit. The rabbit shouted loudly to the turtle to have a race, and the turtle said yes”

## Discussion

Soft palate lengthening using the buccinator myomucosal flap for VPI correction offers several advantages, primarily due to the flap’s high flexibility, which allows for multidirectional rotation and advancement. These characteristics facilitate a tension-free suturing process, thereby simplifying the surgical procedure [3, 13, 14, 16–18]. Moreover, this technique is effectively minimizes the risk of complications such as snoring, mouth breathing, and obstructive sleep apnea, which are more frequently observed with pharyngeal flaps or sphincter pharyngoplasty. The buccinator myomucosal flap is also considered a relatively simple and reliable technique, with low rates of intraoperative and postoperative complications and minimal donor site morbidity. Although rare, postoperative complications such as partial necrosis, partial dehiscence, and donor site hematoma generally resolve spontaneously without additional intervention. Mouth opening limitation may occur even after substantial healing of the operation site, but it is generally manageable with pedicle division under local anesthesia [3, 15]. In this case as well, partial necrosis of the transferred buccal fat flap was observed, but it healed without the need for specific treatment.

Rafael et al. [18] conducted a prospective study on 53 patients who underwent palatal lengthening using bilateral buccinator myomucosal flaps. At 15 months postoperatively, hypernasality, nasal emissions, and intraoral pressure were significantly reduced compared with preoperative levels, with 45 patients (85%) demonstrating successful speech improvement. No cases of complete flap failure, snoring, or sleep apnea were observed in any of the patients.

Monte et al. [19] conducted a retrospective study on 62 non-syndromic VPI patients who underwent palatal lengthening using double opposing buccinator myomucosal flaps. Patients were stratified into three age groups and evaluated over a 15-month postoperative period. Significant improvements were observed in hypernasality, soft palate mobility, and lateral wall motion across all age groups, with no statistically significant differences in outcomes between age groups. Overall, 69.4% of patients demonstrated successful speech improvement, supporting the efficacy of this technique regardless of patient age.

Chiang et al. [20] conducted a retrospective study on 25 patients who underwent secondary palatoplasty using buccal myomucosal flaps for the treatment of velopharyngeal dysfunction (VPD). Speech outcomes were evaluated through perceptual assessments and speech videofluoroscopy. Postoperatively, patients showed a significant increase in velar closing ratio (from 50 to 95%) and improved hypernasality scores (*P* < 0.001). Only 12% of patients had persistent hypernasality, and no cases of obstructive sleep apnea were reported.

The bilateral buccinator myomucosal flap has been established as a reliable technique for palatal lengthening in previous studies [3, 13, 14, 16, 17]. However, reports on the combined use of bilateral buccal fat flaps remain limited. Buccal fat flaps can be easily dissected and positioned between the buccinator myomucosal flaps to reduce dead space, providing a vascular scaffold that may minimize secondary palatal contracture and prevent mucosal flap compromise. Additionally, buccal fat flaps facilitate loose mucosal closure at the base of the buccinator myomucosal flaps, thereby reducing excessive tension on the flap pedicle [16].

In this case report, palatal lengthening was performed using bilateral buccinator myomucosal flaps combined with buccal fat flaps in a 14-year-old female patient with persistent VPI, despite previous primary palatorrhaphy, ongoing speech therapy, and the use of a speech aid. As prior treatments failed to yield significant improvement, hypernasality was determined to be primarily attributable to a short velum rather than muscular dysfunction. Based on this assessment, palatal lengthening was selected over velopharyngeal port narrowing as the surgical approach for VPI correction. Postoperatively, the patient achieved more than 1 cm of soft palate elongation, which was accompanied by notable improvement in hypernasality without complications.

In conclusion, despite the limitation of being a single case with a short follow-up period, the combined use of buccinator myomucosal and buccal fat flaps can be considered a viable treatment option for patients with VPI who suffer from persistent hypernasality due to a short palate. Further research involving larger patient populations is likely to be necessary to confirm these findings and to investigate the potential influence of variables such as patient age, history of speech therapy, and results from other clinical assessments, including magnetic resonance imaging (MRI).

## Data Availability

No datasets were generated or analysed during the current study.

## Abbreviation

VPI: Velopharyngeal insufficiency
